# Application of deep learning-based super-resolution to T1-weighted postcontrast gradient echo imaging of the chest

**DOI:** 10.1007/s11547-022-01587-1

**Published:** 2023-01-07

**Authors:** Simon Maennlin, Daniel Wessling, Judith Herrmann, Haidara Almansour, Dominik Nickel, Stephan Kannengiesser, Saif Afat, Sebastian Gassenmaier

**Affiliations:** 1 Diagnostic and Interventional Radiology, University Hospital Tuebingen, Hoppe- Seyler- Str. 3, 72076 Tübingen, Germany; 2 MR Applications Predevelopment, Siemens Healthcare GmbH, Allee Am Roethelheimpark 2, 91052 Erlangen, Germany

**Keywords:** MRI, Chest imaging, VIBE, Deep learning, Iterative denoising

## Abstract

**Objectives:**

A deep learning-based super-resolution for postcontrast volume-interpolated breath-hold examination (VIBE) of the chest was investigated in this study. Aim was to improve image quality, noise, artifacts and diagnostic confidence without change of acquisition parameters.

**Materials and methods:**

Fifty patients who received VIBE postcontrast imaging of the chest at 1.5 T were included in this retrospective study. After acquisition of the standard VIBE (VIBE_S_), a novel deep learning-based algorithm and a denoising algorithm were applied, resulting in enhanced images (VIBE_DL_). Two radiologists qualitatively evaluated both datasets independently, rating sharpness of soft tissue, vessels, bronchial structures, lymph nodes, artifacts, cardiac motion artifacts, noise levels and overall diagnostic confidence, using a Likert scale ranging from 1 to 4. In the presence of lung lesions, the largest lesion was rated regarding sharpness and diagnostic confidence using the same Likert scale as mentioned above. Additionally, the largest diameter of the lesion was measured.

**Results:**

The sharpness of soft tissue, vessels, bronchial structures and lymph nodes as well as the diagnostic confidence, the extent of artifacts, the extent of cardiac motion artifacts and noise levels were rated superior in VIBE_DL_ (all *P* < 0.001).

There was no significant difference in the diameter or the localization of the largest lung lesion in VIBE_DL_ compared to VIBE_S_. Lesion sharpness as well as detectability was rated significantly better by both readers with VIBE_DL_ (both *P* < 0.001).

**Conclusion:**

The application of a novel deep learning-based super-resolution approach in T1-weighted VIBE postcontrast imaging resulted in an improvement in image quality, noise levels and diagnostic confidence as well as in a shortened acquisition time.

## Introduction

Magnetic resonance imaging (MRI) is an important and frequently used technique for the assessment of thoracic anatomical structures and organs and has been established as an important adjunct to computed tomography [1–3]. A challenge in chest MRI is sufficient robustness to motion due to breathing and heart beat [4]. For this reason, the use of conventional MR sequences such as standard spin-echo sequences is often impractical due to motion caused by the long acquisition time [5]. An established alternative, which promises shorter acquisition times, is a T1-weighted three-dimensional volume-interpolated breath-hold examination (VIBE)-gradient echo (GRE) imaging sequence, which is frequently used in routine clinical practice for contrast-enhanced assessment of thoracic structures [1, 6, 7]. A disadvantage of this sequence is the need of breath holds during image acquisition, which is a major challenge for many patients, especially those with reduced lung function or impaired compliance. There are several approaches to this problem: One commonly used option is respiratory gating [8]. A disadvantage of this approach can be a significantly prolonged examination time [9]. Another common method, which allows a shortening of the necessary breath hold, is the “parallel acquisition technique” (PAT) [10, 11]. The disadvantage of this method is a significant reduction of the signal-to-noise ratio [10]. A possible solution to this problem apart from denoising was demonstrated in a previous study in abdominal imaging via application of a deep learning-based super-resolution algorithm including simulation of acquisition time reduction via partial Fourier technique [12–14].

The aim of this study is to analyze and evaluate the effects of this deep learning-based super-resolution post-processing algorithm for image enhancement and simulation of acquisition time reduction in contrast-enhanced MRI of the chest including PAT.

## Methods

### Study design

This is a retrospective, monocentric study which was approved by the local institutional board. It was conducted in accordance with the ethical standards laid down in the Declaration of Helsinki 1964 as well as in its latest revision 2013. Informed consent of individual subjects was waived.

Fifty patients who underwent a contrast-enhanced MRI of the chest and heart from April 2021 to January 2022 were included in this study. They were identified performing a search using the institutional radiology information system.

### MRI acquisition

MRI acquisition was performed on a 1.5 T MRI scanner (MAGNETOM Aera, Siemens Healthcare, Erlangen, Germany). Patients were in supine position using an 18-channel body coil and 32-channel spine coil. During examination, patients received a bolus of contrast agent according to their body weight with a flow rate of 1.5 ml/s (Gadobutrol, 0.1 mmol/kg, Gadovist, Bayer Healthcare; Leverkusen, Germany). As part of the clinical examination protocol, a postcontrast axial T1-weighted VIBE sequence was acquired three minutes after end of injection using following imaging parameters: repetition time (TR) 6.66 ms, echo times (TE1/TE2) 2.39/4.77 ms, voxel size 1.2 × 1.2 × 3 mm, slice thickness 3 mm, flip angle 10°, matrix size 182 × 320, field of view 317 × 350, parallel imaging factor 4, acquisition time (TA) 16 s, phase partial Fourier 7/8, slice partial Fourier 7/8.

### Retrospective processing using deep learning-based super-resolution and iterative denoising technique

For the evaluation of the employed image enhancement techniques and for the emulation of a more aggressive partial Fourier acquisition, a prototypical processing package was installed on the scanner that allows to perform retrospective reconstructions using the same raw data acquired with the clinical sequence. Since the phase encoding steps of the chosen VIBE protocol are coincidently acquired sequentially in the slice direction, a shorter acquisition corresponding to a slice partial Fourier factor of 6/8 could be simulated by discarding consecutive data acquired at the end of the original acquisition.

In a first step, complex-valued images are reconstructed with the same algorithm as used for the conventional acquisition. Using additional noise information derived from adjustment data available in the scanner integrated reconstruction framework, the images are then denoised with the algorithm detailed in Refs. [13, 14]. In a next step, the images are interpolated by a factor 2 in the phase encoding directions using the super-resolution algorithm outlined in Ref. [12]. Besides super-resolution, the employed network was trained to perform a partial Fourier reconstruction for a slice partial Fourier factor of 6/8 by zero-padding the input to the network in the frequency domain within the supervised training process.

The imaging parameters of the VIBE sequence named above did not change due to the reconstruction process.

### Image evaluation

Image evaluation was performed using a clinical radiological workstation (Centricity PACS RA1000, GE Healthcare, Milwaukee, WI). The VIBE_S_ and VIBE_DL_ images were analyzed by two radiologists with three years of experience in whole body MRI each. Image series were independently evaluated in randomized order and blinded to clinical data. A Likert scale ranging from 1 to 4 was used to evaluate the following criteria: the presence and extent of image artifacts (1, extensive artifacts; 2, severely hampered image quality by artifacts; 3, slightly hampered image quality by artifacts; and 4, no visible artifacts), the presence and extent of image noise (1, extensive noise; 2, severely hampered image quality by noise; 3, slightly hampered image quality by noise; and 4, no visible noise), sharpness of soft tissue borders, bronchial structures, vessels and lymph nodes (1, heavily blurred; 2, severely blurred; 3, slightly blurred; and 4, no blurring with sharp edges). Additionally, myocardial motion artifacts (1, excessive myocardial motion artifacts; 2, severely hampered image quality by myocardial motion artifacts; 3, slightly hampered image quality by myocardial motion artifacts; and 4, no myocardial motion artifacts visible) and diagnostic confidence (1, non-diagnostic; 2, poor image quality; 3, good image quality; and 4, excellent image quality) were evaluated.

In case of the presence of visible lung lesions, the largest lesion per patient was evaluated, measuring its largest diameter as well as again using a Likert scale 1 to 4 to assess lesion detectability (1, lesion border not detectable; 2, poor detectability of lesion border; 3, good detectability of lesion boarder; and 4 excellent detectability of lesion border).

### Statistical evaluation

Statistical analysis was performed using statistical software (SPSS Statistics Version 28, IBM; Armonk, NY). Parametric data are depicted using mean ± standard deviation. Nonparametric data are depicted using median and interquartile ranges. Wilcoxon signed-rank test was applied to compare the ordinal-scaled data between VIBE_S_ and VIBE_DL_. Linearly weighted Cohen κ was used to calculate interreader agreement. *P* values below 0.05 were assumed to be significant.

## Results

### Patient cohort

Image data of all fifty patients were evaluated successfully. Mean patient age was 44 ± 18 years, comprising a range from 18 to 84 years. Most frequently stated clinical indication for MRI was the question of possible myocarditis. In 25 cases, at least one lung lesion could be detected. Further information is depicted in Table 1.

**Table 1:** Patient’s characteristics

Characteristics	
Patients, n	50
Age, mean ± SD (range), *y*	44 ± 18 (18–84)
Indication for examination, *n*	
Amyloidosis	3
Cardiomyopathia	4
Arrhythmia	4
Myocardial infarction	7
Myocarditis	23
Pericarditis	2
Sarcoidosis	1
Systemic sclerosis	2
Cardiac thrombus	1
VSD	1
Turner-Syndrom	1
Pulmonal lesions, *n*	
No finding	25
Nodule	22
Abscess	1
Dystelectasis	1
Alveolitis	1

### Interreader variability

Interreader agreement was almost perfect regrading evaluation of image quality as well as lung lesions as Cohen *κ* was 0.82. Therefore, only the results of reader 1 are depicted in the following. The results of reader 2 are shown in Tables 2 and 3.

**Table 2:** Evaluation of image quality and diagnostic confidence

	Reader 1			Reader 2		
Characteristics	VIBE_S_	VIBE_DL_	*P*	VIBE_S_	VIBE_DL_	*P*
Artifacts	3 (3–3)	3 (3–4)	< 0.001	3 (3–3)	3 (3–4)	< 0.001
Noise	3 (2–3)	4 (3–4)	< 0.001	2 (2–3)	3 (3–4)	< 0.001
Sharpness soft tissue	3 (3–3)	4 (4–4)	< 0.001	3 (3–3)	4 (4–4)	< 0.001
Sharpness bronchial structures	3 (2.75–3)	4 (4–4)	< 0.001	3 (2–3)	4 (3.75–4)	< 0.001
Sharpness vessels	3 (3–3)	4 (4–4)	< 0.001	3 (2.75–3)	4 (4–4)	< 0.001
Sharpness lymph nodes	3 (2.75–3)	4 (4–4)	< 0.001	3 (2–3)	4 (4–4)	< 0.001
Motion artifacts myocardium	3 (3–3)	4 (3–4)	< 0.001	3 (3–3.25)	4 (3–4)	< 0.001
Diagnostic confidence	3 (3–3)	4 (4–4)	< 0.001	3 (3–3)	4 (4–4)	< 0.001

*VIBE* volume-interpolated breath-hold examination, *VIBE*_*S*_ standard VIBE datasets, *VIBE*_*DL*_ denoised datasets

**Table 3:** Evaluation of image quality and diagnostic confidence of lung lesions

	Reader 1			Reader 2		
Characteristics	VIBE_S_	VIBE_DL_	*P*	VIBE_S_	VIBE_DL_	*P*
Sharpness lung lesions	2 (2–2)	3 (3–3)	< 0.001	2 (2–2)	3 (3–3)	< 0.001
Lesion detectability	2 (2–2)	3 (3–3)	< 0.001	2 (2–2)	3 (3–3)	< 0.001

*VIBE* volume-interpolated breath-hold examination, *VIBE*_*S*_ standard VIBE datasets, *VIBE*_*DL*_ denoised datasets

### Acquisition time

The mean acquisition time for one VIBE_S_ sequence was 15.2 ± 1.0 s. As the employed VIBE protocol acquires phase encoding steps sequentially in the slice direction, an acquisition with a higher partial Fourier factor in the slice direction can be simulated by omitting the corresponding data at the end of the acquisition. This was implemented for a partial Fourier factor of 6/8 in the slice direction, corresponding to the training of the employed deep learning-based super-resolution network. The mean simulated acquisition time for one VIBE_DL_ sequence was 13.1 ± 0.8. This way, a reduction in acquisition time of 2.1 s on a mean could be achieved (*P* < 0.001).

### Qualitative evaluation of image quality

The sharpness of soft tissue as well as of bronchial structures, vessels and lymph nodes was rated significantly higher in VIBE_DL_ as in VIBE_S_. Accordingly, the sharpness of soft tissue improved from a median of 3 (3–3) to 4 (4–4) (*P* < 0.001), the sharpness of bronchial structures from a median of 3 (2.75–3) to 4 (4–4) (*P* < 0.001), the sharpness from vessels was rated improved, from a median of 3 (3–3) to 4 (4–4) (*P* < 0.001) as well as the sharpness of lymph nodes (from a median of 3 (2.75–3) to 4 (4–4), *P* < 0.001).

In comparison with VIBE_S_, VIBE_DL_ was also rated with reduced noise with a median of 4 (3–4) versus 3 (2–3) (*P* < 0.001) and less severe artifacts with a median of 3 (3–4) compared to 3 (3–3) (*P* < 0.001). Additionally, myocardial motion artifacts were also found reduced for VIBE_DL_ as the median increased from 3 (3–3) to 4 (3–4) (*P* < 0.001).

Overall, the diagnostic confidence of the images was rated improved for VIBE_DL_ compared to VIBE_S_ as the median increased from 3 (3–3) to 4 (4–4).

**Fig. 1:**
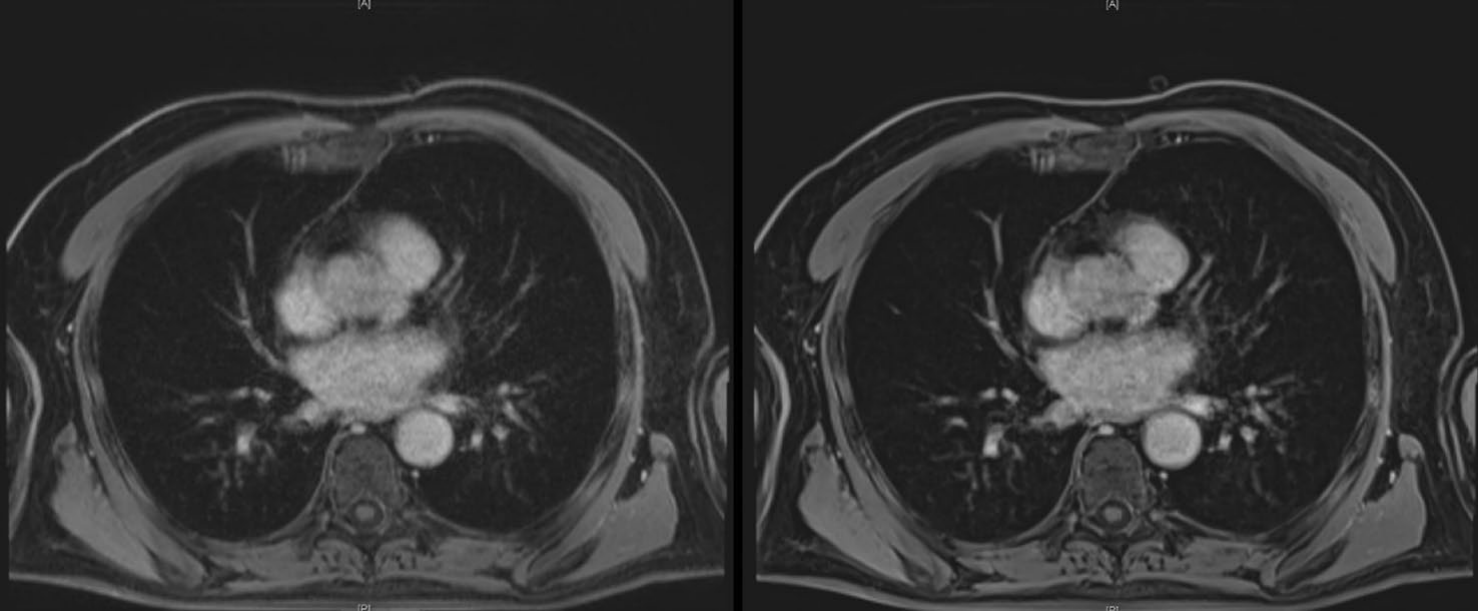
Comparison of reconstructions for an exemplary dataset. On the left side, the conventional reconstruction (VIBE_S_) is depicted, whereas the right side shows the reconstruction using super-resolution, denoising and a simulated partial Fourier acquisition (VIBE_DL_). The VIBE_DL_ dataset shows reduced noise and artifacts with enhanced sharpness of anatomical structures, e.g., the vessels

Figure 1 shows exemplary images for VIBE_s_ and VIBE_DL_.

### Comparison of lung lesions

Of the fifty enclosed patients, 25 showed at least one lung lesion in the acquired images. As depicted in Table 1, in 23 patients at least one lung nodule was found. In one case, the largest detectable lung lesion was an abscess; in another case the largest lesion occurred in the setting of an alveolitis and in one case in the setting of dystelectasis.

Qualitative evaluation of lesion sharpness and detectability demonstrated a significant improvement in VIBE_DL_ with a median of 3 (3–3) as compared to a median of 2 (2–2) in VIBE_S_ for both categories and both readers (*P* < 0.001). The results are shown in Table 3.

There could not be found a significant difference in lesion size comparing VIBE_DL_ to VIBE_S_ for both readers as depicted in Table 4. Reader 1 measured a median lung lesion diameter of 4 mm (2–5.5) for VIBE_DL_ as well as for the standard VIBE (*P* = 0.157). Reader 2 measured a median lesion diameter of 4 mm (2–6) for VIBE_S_ and 4 mm (2–5.5) for VIBE_DL_ (*P* = 0.705).

**Table 4:** Evaluation of lung lesion diameter in millimeter

	Reader 1			Reader 2		
Characteristics	VIBE_S_	VIBE_DL_	*P*	VIBE_S_	VIBE_DL_	*P*
Lesion diameter	4 (2–5.5)	4 (2–5.5)	0.157	4 (2–6)	4 (2–5.5)	0.705

*VIBE* volume-interpolated breath-hold examination, *VIBE*_*S*_ standard VIBE datasets, *VIBE*_*DL*_ denoised datasets

**Fig. 2:**
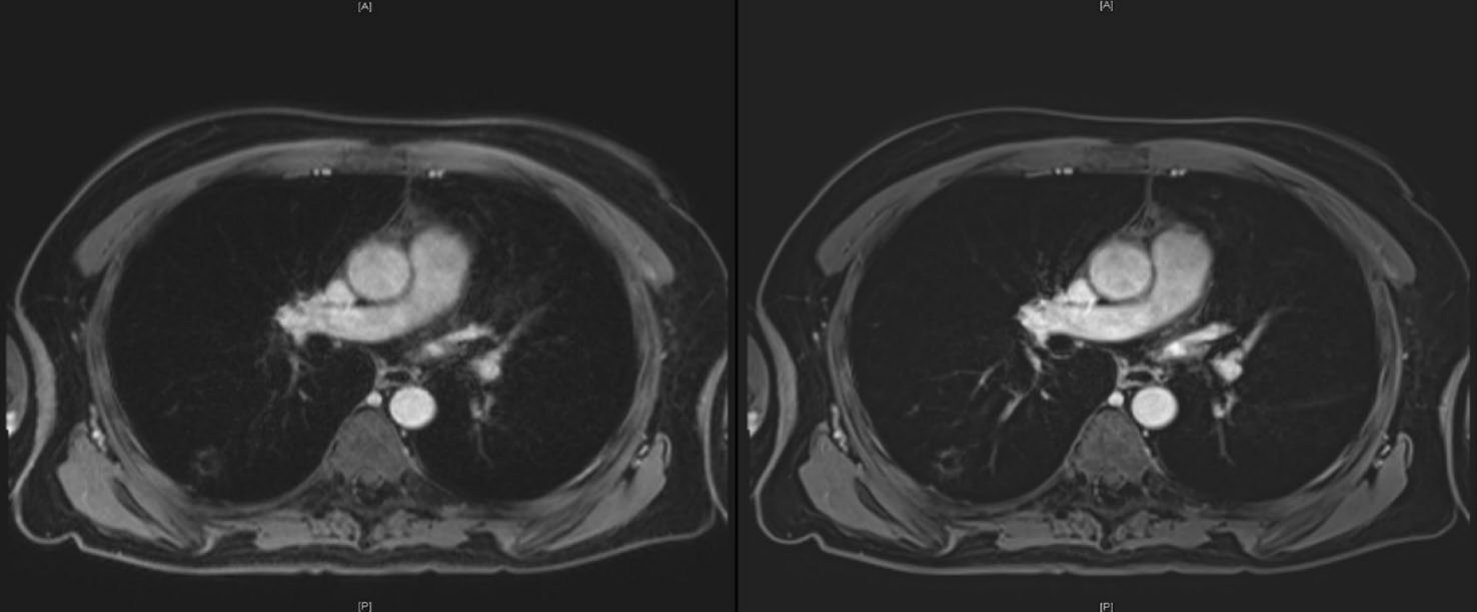
Comparison of reconstructions for an exemplary dataset. On the left side, the conventional reconstruction (VIBE_S_) is depicted, whereas the right side shows the reconstruction using super-resolution, denoising and a simulated partial Fourier acquisition (VIBE_DL_). In the right upper lobe, a lung lesion was detected which was diagnostically classified as an abscess. In the VIBE_DL_ image, the lesion shows enhanced sharpness and contrast in comparison with the VIBE_S_ image

Figure 2 shows exemplary pictures of a lung abscess in the right upper lung lobe for VIBE_S_ and VIBE_DL_.

## Discussion

A deep learning-based super-resolution approach combined with iterative denoising and simulating a more aggressive partial Fourier acquisition corresponding to a shorter acquisition was evaluated in this study analyzing fifty postcontrast data sets of the chest acquired with T1-weighted VIBE imaging. Assessing the enhanced VIBE images in comparison with the standard VIBE, improved sharpness of various thoracic structures, decreased noise levels, reduced severe artifacts and an improved diagnostic confidence were found.

Conventional Cartesian T1-weighted GRE-VIBE sequences are one of the standard MR sequences used to assess thoracic structures and pathologies [6, 15]. GRE imaging enables prompt and high-resolution image acquisition in clinical routine as wells as in research. A major advantage of GRE imaging in comparison with spin-echo or turbo-spin-echo imaging is the considerable reduction of motion artifacts [16]. In contrast, a crucial disadvantage of GRE imaging is the enhanced vulnerability to susceptibility artifacts in the presence of magnetic field inhomogeneities [16]. Yet another disadvantage of Cartesian VIBE imaging that comes into play is the need for breath holds, especially when acquiring images of the thorax. This issue comes particularly into display when examining severely ill persons and children or elderly patients.

A possible approach to respond to these challenges is the application of a free breathing 3D-ultra-short echo time (UTE) VIBE sequence as demonstrated by Olthof et al. [17]. Another possibility is the usage of a radial GRE sequence with k-space-weighted image contrast (KWIC) reconstruction [18]. However, major disadvantage of these techniques is the considerable increase in acquisition time. Additionally, a reduced vessel-tissue contrast was reported for the usage of radial readout instead of Cartesian readout [19].

An often applied technique to decrease acquisition time as well as minimize required breath-hold time is parallel imaging [20]. An important drawback of sufficient parallel imaging to reduce breath-hold time, however, is the need for high parallel imaging factors which leads to a decrease in image quality and SNR. Apart from denoising only, deep learning-based methods have been discussed in the past to tackle this issue including acquisition time reduction [12].

Therefore, the aim of this study was to demonstrate the feasibility and reliability of the deep learning-based super-resolution reconstruction approach to reduce noise and improve image quality in VIBE imaging of the chest. Additionally, the occurrence and extent of artifacts could be reduced in VIBE_DL_ compared to VIBE_S_ as shown in Fig. 1. As depicted above, we found an enhanced sharpness of thoracic structures when applying the modified reconstruction approach. There could be also observed a reduction of motions artifacts, in particular cardiac motion artifacts.

An advantage of the evaluated approach is its simplicity in usage. The workflow can be implemented in the scanner architecture without the need to alter already established and used workflows. Further decrease in acquisition time in future might be possible via simulation of more aggressive partial Fourier settings.

Furthermore, this work outlines the potentials of deep learning image reconstruction which will have an enormous impact on radiological workflows in future. The advantages regarding image reconstruction, acquisition time reduction as well as image quality improvement have been demonstrated in various fields, especially in abdomino-pelvic and musculoskeletal imaging [21–25]. In chest imaging, applications of deep learning mostly concentrated on cardiac imaging in particular or computed tomography of the chest [26–29]. Therefore, this present study could successfully highlight the benefits of super-resolution applications in MR chest imaging. This can be of outmost importance in the development of MR protocols of the lungs in young patients in need of routine monitoring of the lungs due to chronic diseases, such as cystic fibrosis [30].

We acknowledge several limitations of our study. One fact to consider is the lack of quantitative comparison of noise levels, respectively, SNR of VIBE_DL_ compared to the VIBE_S_. Further software development will be necessary to enable quantitative analysis of SNR.

Additionally, the feasibility of this method was investigated on a 1.5 T MRI only. Hence, there is no experience with using this approach in higher field strengths. Another limitation is the limited number of included patients. Besides, this study had a retrospective study design. Therefore, the acquisition protocol was designed for clinical purposes and the VIBE_DL_ images were calculated retrospectively.

In conclusion, a significant improvement of image quality, noise and diagnostic confidence was achieved using a novel deep learning-based super-resolution technique in T1-weighted VIBE postcontrast imaging of the chest. In future, this finding could lead to a further decrease in acquisition time without the loss of image quality.

## Data Availability

The datasets generated during and/or analyzed during the current study are available from the corresponding author on reasonable request.
